# Extensive polyploid clonality was a successful strategy for seagrass to expand into a newly submerged environment

**DOI:** 10.1098/rspb.2022.0538

**Published:** 2022-06-08

**Authors:** Jane M. Edgeloe, Anita A. Severn-Ellis, Philipp E. Bayer, Shaghayegh Mehravi, Martin F. Breed, Siegfried L. Krauss, Jacqueline Batley, Gary A. Kendrick, Elizabeth A. Sinclair

**Affiliations:** ^1^ School of Biological Sciences, University of Western Australia, Crawley, Western Australia, 6009, Australia; ^2^ Oceans Institute, University of Western Australia, Crawley, Western Australia, 6009, Australia; ^3^ Kings Park Science, Department of Biodiversity Conservation and Attractions, 1 Kattidj Close, West Perth, Western Australia 6005, Australia; ^4^ College of Science and Engineering, Flinders University, Bedford Park, South Australia 5042, Australia

**Keywords:** ddRAD-seq, flow cytometry, heterozygosity, karyotyping, population genomics, *Posidonia australis*

## Abstract

Polyploidy has the potential to allow organisms to outcompete their diploid progenitor(s) and occupy new environments. Shark Bay, Western Australia, is a World Heritage Area dominated by temperate seagrass meadows including Poseidon's ribbon weed, *Posidonia australis*. This seagrass is at the northern extent of its natural geographic range and experiences extremes in temperature and salinity. Our genomic and cytogenetic assessments of 10 meadows identified geographically restricted, diploid clones (2*n* = 20) in a single location, and a single widespread, high-heterozygosity, polyploid clone (2*n* = 40) in all other locations. The polyploid clone spanned at least 180 km, making it the largest known example of a clone in any environment on earth. Whole-genome duplication through polyploidy, combined with clonality, may have provided the mechanism for *P. australis* to expand into new habitats and adapt to new environments that became increasingly stressful for its diploid progenitor(s). The new polyploid clone probably formed in shallow waters after the inundation of Shark Bay less than 8500 years ago and subsequently expanded via vegetative growth into newly submerged habitats.

## 1. Introduction

Whole-genome duplication through polyploidy is a widely repeated mechanism of significant diversification throughout the evolutionary history of flowering plants [1–6]. Phylogenomic analyses now suggest all angiosperms have been through at least one round of polyploidization [7]. Past polyploid events appear to be associated with periods of significant changes in global climate [5], including deglaciation of terrestrial environments since the Last Glacial Maximum (e.g. [8,9]). Terrestrial polyploids are widespread globally, but more frequent at higher latitudes and in extreme environments [10]. They are also frequently found in habitats that contrast to the habitat of their diploid progenitor(s), indicating polyploidy is associated with evolutionary success in terms of their ability to occupy new environmental niches [11–13].

In a global review of terrestrial ecosystems, specific environmental and life-history attributes were highlighted by [10] that facilitate the establishment of new polyploid lineages. The attributes provide polyploids with sufficient time (usually perennial species with vegetative growth) and space (new environments with low species richness) to outcompete their diploid progenitor(s). Polyploidy has often been regarded as an ‘evolutionary dead end’, yet the process can create ‘hopeful monsters’ by conferring a rapid increase in genetic diversity that can facilitate an adaptive advantage over diploid progenitor(s), especially under environmentally stressful conditions [5,6,14,15]. The success of genome duplication depends strongly on morphological, physiological and ecological attributes of the new polyploid, with ploidy-related changes also influencing species interactions through gene expression and epigenetic processes (reviewed in [5,16]). Has polyploidy also played a role in diversification and expansion of marine angiosperms—seagrasses?

Seagrasses are a polyphyletic group which evolved through at least three independent ‘return to the sea’ events [17] in the early Cretaceous [4]. Seagrasses now inhabit marine coastlines and estuaries globally, except for Antarctica [18]. They reproduce sexually through flowering and seed production, and clonally through vegetative growth via horizontal rhizome extension [19]. Most seagrass species have broad geographical distributions with wide-ranging levels of genetic diversity (e.g. [20,21]), and some meadows have expansive, long-lived clones (e.g. [22,23]). Additional alleles at co-dominant genetic markers (3+ alleles) have been reported in several seagrass species [24,25], including Poseidon's ribbon weed, *Posidonia australis,* in Shark Bay [26,27], and are indicative of polyploidy. Variation in chromosome number among populations has been reported in several seagrass species [28,29] including *Cymodocea angustata* in Shark Bay, Western Australia [29]. The possibility of hybridization was first identified in the genus *Zostera* [30], and more recent studies indicate that phenomenon is probably more widespread in seagrasses [31–35].

**Figure 1.**
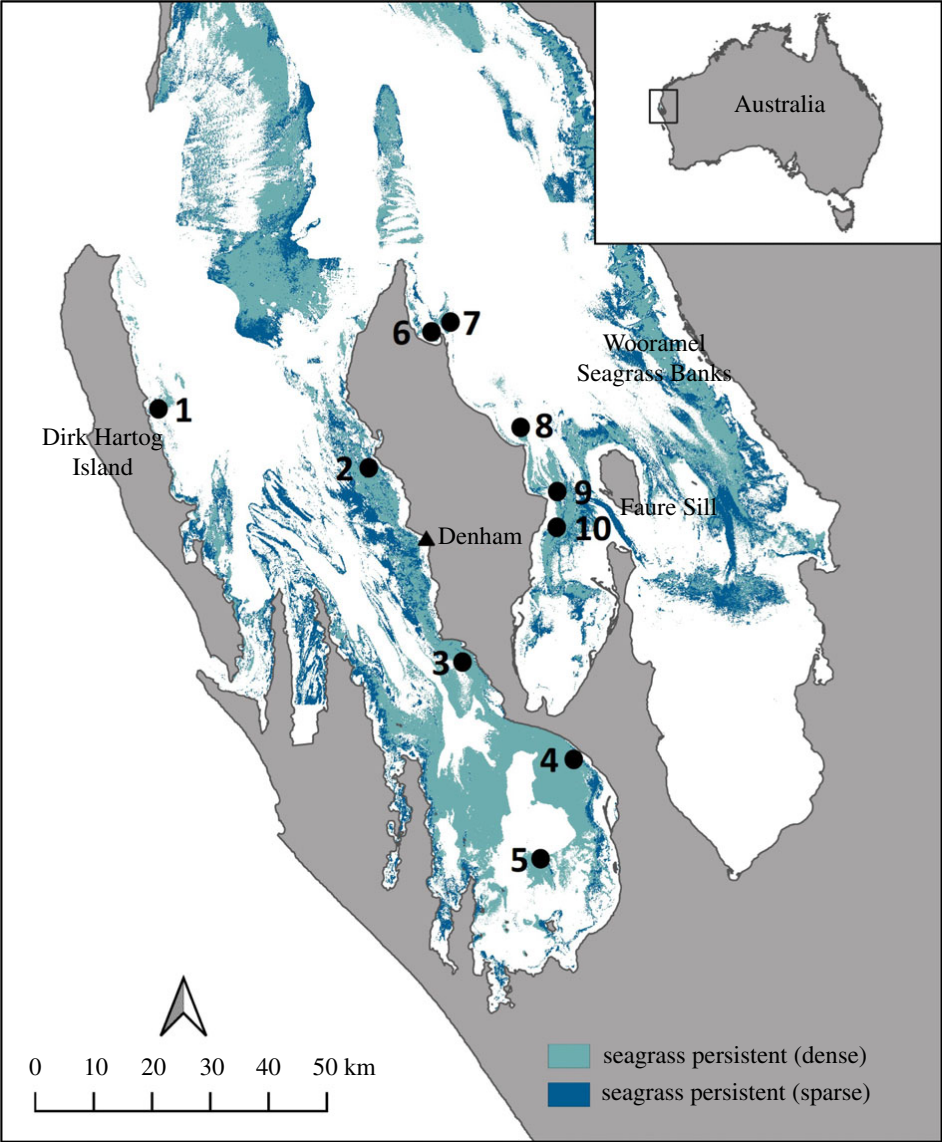
Map of Shark Bay, Gathaagudu, Western Australia. Distribution of persistent seagrass cover (dense and sparse) in 2016 (adapted from Strydom *et al*. [37]). *Posidonia australis* sampling locations for meadows in the western gulf (1, Sandy Point, Dirk Hartog Island; 2, Middle Bluff; 3, Fowlers Camp; 4, Nanga Bay; 5, White Island) and eastern gulf (6, Herald Bight; 7, Guischenault Point; 8, Monkey Mia; 9, Dubaut Point; 10, Faure Sill). (Online version in colour.)

Seagrasses recolonized the Australian Continental shelf with rising sea levels following the Last Glacial Maximum [36], and this included the marine transgression at Shark Bay (figure 1) [38]. A period of rapid sea-level rise during the Holocene (1–2 cm year^−1^; see [39] and references therein) created extensive inundation and new habitat for benthic marine species, including temperate seagrasses. Expanding seagrass meadows trap sediments which ultimately control environmental gradients through the development of the Faure Sill and Wooramel Seagrass Bank [38], creating increasingly extreme environments for seagrasses and other marine species to inhabit. The metahaline and hypersaline shallow waters within Shark Bay now experience temporal and spatial fluctuations in temperature and salinity in phosphorus-limited waters [40–42]. Here, we use a genotype-by-sequencing approach to (i) assess population genetic diversity and structure of *P. australis* across the environmental gradient within Shark Bay and (ii) use karyotyping and flow cytometry to determine the presence of polyploidy.

## 2. Material and methods

### (a) Shark Bay and temperate seagrasses

The UNESCO World Heritage Site of Shark Bay, or Gathaagudu to the traditional custodians, the Malgana Peoples, is home to one of the largest continuous seagrass meadows in the world and creates habitat for a biodiverse marine environment including 12 species of seagrass (figure 1) [43]. A horizontal salinity gradient varies with tidal wave propagation across the shallow bays and sills diurnally, seasonally and varies by gulf [44]. A combination of minimal freshwater input, poor-flushing and high evaporation (approx. 2000 mm) which exceeds precipitation (approx. 200 mm) [37,45] maintains a steep gradient in the eastern gulf. The clear, shallow waters across most of the Bay mean that seagrass meadows are exposed to saturated light levels (greater than 3000 µmol m s^−1^), experience a large annual range in temperature, typically 17–26°C although temperatures can exceed 30°C in summer [42], and a salinity range between 35 (oceanic) and 64 psu [43]. Large, perennial, seagrass meadows of *Amphibolis antarctica* and *Posidonia australis* dominate much of this marine ecosystem [43], creating an ideal location in which to study evolutionary change and adaptation.

Shark Bay is situated at the temperate–tropical interface meaning it is exposed to temperate and tropical extremes of climate change and extreme weather events, such as marine heatwaves and cyclones. The west coast of Australia was exposed to an unprecedented heatwave in the summer of 2010–2011, which impacted both terrestrial and marine ecosystems [46], with sea surface temperatures greater than 3°C above long-term averages in Shark Bay [47]. Most areal loss of seagrass meadows was associated with *Amphibolis antarctica,* where extensive defoliation resulted in local extinction of meadows [48]. A total estimated area of 1310 km^2^ of dense seagrass meadows disappeared between 2010 and 2014 and consisted almost entirely of temperate species [37]. The approximately 200 km^2^ of *Posidonia australis* meadows were also impacted, although some natural recovery has occurred where shoot densities have returned to pre-heatwave levels in some locations [49].

### (b) Genetic sampling and laboratory protocols

Adult shoot samples of *P. australis* were collected via SCUBA in 2012 and 2019 from 10 meadows across the geographic range within Shark Bay (figure 1; electronic supplementary material, table S1). Randomized shoot sampling was standardized across sites (within a 50 m diameter area) following [50]. Shoot meristem (non-photosynthetic) tissue was processed and frozen prior to DNA extraction, as described in [51]. Local environmental conditions measured *in situ* at the time of sampling include depth (m), water temperature (°C), salinity (practical salinity units, psu) and pH.

Genomic DNA was extracted from frozen shoot meristems using a Qiagen DNeasy Plant Pro Kit (Qiagen, Germany) for 144 samples from 10 sampled meadows (12–14 per meadow). Manufacturer protocols were used with the following modifications to improve DNA quality and quantity recovered: half a frozen shoot meristem (approx. 20 mm, approximately 0.25 g) was ground in a mortar and pestle to a fine powder in liquid nitrogen. A buffer mix of 900 µl of CD1 and 100 µl of PS solution was added to the ground sample and left to defrost. The defrosted sample was transferred into a 2 ml microcentrifuge tube containing one ceramic bead and 3 µl of *Rnase.* The tubes were placed on a shaker for 10 min, followed by centrifugation at 16 128 RCF for 2 min. The supernatant was transferred into a clean labelled 1.5 ml collection tube. Four hundred microliter of CD2 was added to the supernatant, vortexed for 5 s and placed on ice for 5 min. The final genomic DNA was suspended in 50 µl of EB buffer, which was soaked on the spin column membrane for 30 min. The genomic DNA samples were stored at −20°C. Genomic DNA quantity was measured using the dsDNA broad range Qubit 3.0 Fluorometer (ThermoFisher Scientific, Australia) and the quality was assessed using the Lab Chip GX Touch 24 (PerkinElmer) with HT DNA gDNA reagents.

Library preparation followed the protocol for ddRAD-seq in [52]. Fourteen samples were genotyped from each meadow, with four technical replicates included. Samples were randomly assigned across three libraries, with no two pairs of replicates occurring in the same library. Pooled libraries were sequenced on a HighSeqX10 sequencing machine as 2 × 150 bp paired-end reads (KCCG Sequencing Laboratory, Garvan Institute, New South Wales). Raw reads were processed following the pipeline detailed in [52]. In brief, a *de novo* ddRAD loci assembly and identification of single-nucleotide polymorphisms (SNPs) was performed using the *denovo_map* pipeline in STACKS v. 2.52 [53]. A minimum distance of three nucleotides was used to identify a stack (-m), a maximum of three nucleotides were permitted between stacks (-M) and a maximum of three mismatches were permitted between loci of different individuals during catalogue construction (-n) (electronic supplementary material, table S2). A total of 133 out of 144 samples remained after the m3-M3-n3 pipelining was completed. We used read depth to estimate a new statistic, *H*_ind_/*H_E_* [54] to identify individuals with unexpected ploidy and hybrid status. The expected value of *H*_ind_/*H_E_* is the same across all loci in a dataset, regardless of read depth or allele frequency. The *H*_ind_*/H_E_* statistic was calculated for all samples, where a *H*_ind_*/H_E_* of 0.50 is expected for diploid individuals, 0.75 for triploids, and 1.00 for tetraploids. The statistic was estimated using PolyRAD v. 1.5 (available at https://github.com/lvclark/polyRAD).

### (c) Genomic diversity and structure

Sequencing error was assessed by comparing the percentage of non-identical SNP alleles across the four pairs of technical replicates. An error rate cut-off was based on the maximum difference between the four pairs of technical replicates. Percentage similarity was determined as a measure of the variation among SNP profiles within each of the 10 sampled meadows. Technical replicates were removed prior to all subsequent analyses. Clonal richness (*R* = (MLG − 1)/(*N* − 1)) [55] was assessed after applying an error rate estimated from the technical replicates using the R package Poppr [56], with values close to zero indicating high levels of clonality and 1 indicating all samples were from different plants. Diversity statistics were calculated based on all SNPs and estimated using Stacks: populations within the pipeline (https://github.com/ascheben/RAD_analysis_workflow#Diversity-analysis-protocol [52]. These diversity statistics included number of private alleles in the population (private, alleles that occur only in a single sample location), mean frequency of the most common allele at each locus (P), observed (*H*_o_) and expected heterozygosity (*H*_e_), inbreeding coefficient (*F*_IS_) and nucleotide diversity (*π*). A cladogram was generated to visualize the relationship among all individuals using (SNPRelate 1.28.0 [57]).

An assessment of population structure was conducted using the whole dataset (*n* = 10 meadows, 18 021 SNPs). Identification of the extent of admixture among sampled *P. australis* meadows estimated ancestry proportions using an approach based on sparse non-negative matrix factorization (sNMF) [58]. The sNMF program assumes that genetic data originates from the admixture of *K* parental populations, where *K* is unknown [58]. An estimate of ancestry proportions for each sample was computed. The number of distinct genetic clusters (*K*) was determined, based on 10 iterations per *K* value for *K* = 1 to 10 for the complete dataset, and for nine high heterozygosity meadows *K* = 1 to 9. Cross-entropy plots were generated to visualize the optimal number of *K* ancestral populations. A hierarchical analysis of molecular variation (AMOVA) was performed to partition genetic diversity by ploidy and within and among high heterozygosity meadow using all SNPs using Poppr. Variance components were computed at multiple levels to test for significance between ploidy, among meadows and within meadows, with significance based on 9999 permutations.

### (d) Estimation of genome size using flow cytometry

Nuclear DNA content was estimated using tomato (*Solanum lycopersicum* cv. Stupick, 2C DNA = 1.96 pg) as an internal size standard. *Posidonia australis* nuclei were isolated from healthy fresh young leaves by chopping with a sharp razor blade in 1 ml of woody plant buffer [59] in a Petri dish, supplemented with 50 µl of propidium iodide (PI; 1 mg ml^−1^, Sigma) and RNase A (50 µg ml^−1^). The nuclear suspension was then filtered through a 30 µm nylon mesh filter and analysed using a BD FACSCanto flow cytometer equipped with a high-grade solid state laser with green light emission at 488 nm, operating at 20 mW, as well as with side (SSC) and forward (FSC) scatters. Analyses were performed on three different samples from each population. Histograms with a coefficient of variation lower than 5% were evaluated using the FloJo program [60]. Monoploid genome size (1 C*x*) was calculated based on a conversion factor [61], where 1 pg of DNA content represents 978 mega base pairs.

### (e) Determination of ploidy via karyotyping

Fresh root tips were available for plants from two sites, Guischenault Point and Middle Bluff (sites 2, 7; figure 1). Root tips were pre-treated in 2 mM 8-hydroxyquinoline (4 h) and then fixed in freshly prepared ethanol: glacial acetic acid (3 : 1, v/v) for 30 h, and finally stored in the same solution at −20°C until use. Chromosome preparations from root tip were carried out as described in [62]. Five mitotic metaphases were selected at random from each sample and washed twice in ice cold water (5 min each time), followed by 0.01 M citrate buffer (0.01 M citric acid and 0.01 M sodium citrate, pH 4.8) twice for 5 min. Root tips were then digested in 30 µl enzyme mixture (including 0.7% cellulose, 1% cytohelicase, 1% pectolyase and 0.7% cellulose R10) for 60–90 min. After digestion, meristems were washed twice with citrate buffer and once with ethanol. Ethanol was replaced with 60–90 µl freshly prepared fixative (9 : 1, absolute glacial acetic acid : absolute methanol). The meristems were carefully broken using a needle to obtain cell suspension. Seven microlitre of the cell suspension was dropped onto each glass slide in a box lined with wet paper towels (to have about 50% humidity inside the box) and left to dry slowly. Slides were dehydrated in ethanol series, dried at room temperature and mounted in 1 µg ml^−1^ DAPI (4′,6-diamidino2-phenylindole) as counterstain. Slides were analysed with a confocal microscope, and images were captured. A total of eight chromosomal parameters and karyotypic (or asymmetric) indices were measured in triplicate for Guischenault Point and Middle Bluff plants using the computer software *IdeoKar* [63]. These include length of the long chromosome arm (*L*), length of the short chromosome arm (*S*), chromosome length (CL = *L* + *S*), arm ratio (AR = *L*/*S*), *r*-value (=*S*/*L*); relative length of chromosome (RL), form percentage of chromosome (F%), centromeric index (CI = *S*/CL) and DNA C-value = DNA 2C-value (pg). Two-tailed tests were performed to determine whether there was a significant difference between the eight chromosomal parameters and karyotypic indices between Guischenault Point and Middle Bluff.

## 3. Results

### (a) Raw sequence filtering, error rate and coverage

A total of 133 samples including four technical replicates from 10 meadows were sampled across Shark Bay and remained after the removal of 11 samples which did not pass the filtering parameters (missingness greater than 90% on a read depth of 5 and a minimum minor allele filtering; MAF = 0.05; electronic supplementary material, table S2). The complete SNP dataset consisted of 18 021 biallelic SNPs after filtering. The number of SNP differences between each of the four pairs of technical replicates ranged between 355 and 439 (out of 15 625–16 660 SNPs allowing for missing data), which equated to a maximum difference of 2.8%. All mismatches between SNP loci occurred where one individual was homozygous and one was heterozygous, except three SNPs, which were homozygous for alternate alleles. Technical replicates were removed for all subsequent statistical and diversity estimates, and we applied a maximum error rate of 2.8% to estimate clonal richness. The distribution of depth coverage was similar across all samples (*H*_ind_/*H_E_* greater than 0.8), except those from Guischenault Point where *H_i_*_nd_/*H_E_* less than 0.5 (electronic supplementary material, figure S1*a*). The overall weighted mean *H*_ind_/*H_E_* was 0.95 when including Guischenault Point individuals and 1.0 when excluding Guischenault Point individuals (electronic supplementary material, figure S1*b*).

### (b) Genetic diversity and population genetic structure

Diversity statistics were remarkably similar for nine out of 10 sampled meadows (table 1). Almost identical multi-locus SNP profiles (=multi - locus genotypes) were identified across these nine meadows which spanned the entire 200 km^2^ of mapped *P. australis* meadows in Shark Bay, over a wide salinity range (33.9–48.8 psu; electronic supplementary material, table S1). One widespread multi-locus genotype was shared among seven out of nine meadows (sites 2, 3, 4, 6, 8, 9 and 10), a northern multi-locus genotype was present in Dirk Hartog Island and Herald Bight (sites 1 and 6), a southwest multi-locus genotype was from Nanga Bay and White Island (sites 4 and 5), and a low-frequency multi-locus genotype (two samples) was unique to Fowlers Camp (site 3). The nine meadows were highly heterozygous (mean *H*_o_ = 0.891 ± 0.078 s.d.), with high-nucleotide diversity (*π* = 0.477 ± 0.005 s.d.) and a high percentage SNP identity (mean identity = 0.958 ± 0.008 s.d.). The highly negative *F*_IS_ values (mean *F*_IS_ = −0.801 ± 0.012 s.d.) indicated an excess of observed heterozygotes.

**Table 1.** Population diversity statistics. Diversity statistics for 10 sampled *P. australis* meadows based on 18 021 SNPs: *N* = number of samples sequenced; unique profile = number of unique multi-locus SNP profiles; *R* = clonal diversity; private = number of private alleles; *P* = frequency of the most common allele at each locus; *H*_o_ (%) = observed heterozygosity; *H*_e_ (%) = expected heterozygosity; *F*_IS_ = inbreeding coefficient; *π* = nucleotide diversity; SNP identity = proportion of shared SNPs (±s.d.).

pop	meadow	abbrev.	*n*	unique profile	*R*	private	*p*	*H*_o_ (%)	*H*_e_ (%)	*F*_IS_	*π*	SNP identity (±s.d.)
1	Dirk Hartog	DH	14	1	0.00	87	0.547	90.5	46.2	−0.814	0.482	0.960 ± 0.004
2	Middle Bluff	MB*	12	1	0.00	0	0.555	88.5	45.4	−0.787	0.475	0.959 ± 0.006
3	Fowlers Camp	FC	12	2	0.09	1	0.558	88.0	45.3	−0.783	0.473	0.953 ± 0.008
4	Nanga Bay	NB	14	2	0.08	0	0.551	89.4	46.2	−0.806	0.478	0.944 ± 0.016
5	White Island	WH	13	1	0.00	0	0.542	89.5	46.6	−0.821	0.486	0.966 ± 0.003
6	Herald Bight	HB	14	2	0.08	0	0.551	89.6	46.1	−0.806	0.479	0.949 ± 0.019
7	Guischenault Point	GU*	13	8	0.58	207	0.819	24.6	23.0	0.016	0.241	0.735 ± 0.129
8	Monkey Mia	MM	13	1	0.00	14	0.556	88.5	45.4	−0.797	0.472	0.960 ± 0.004
9	Dubaut Point	DP	12	1	0.00	0	0.551	89.6	45.7	−0.801	0.478	0.966 ± 0.005
10	Faure sill	FI	12	1	0.00	24	0.556	88.6	45.1	−0.793	0.472	0.970 ± 0.002
average over nine high heterozygosity meadows:			116	4	0.02	—	0.552	89.1	45.8	−0.801	0.477	0.958 ± 0.008

*Ploidy determined.

Diversity statistics for the Guischenault Point meadow (site 7) were very different to all other meadows (table 1). Eight unique multi-locus genotypes were identified from the reproductive meadow at Guischenault Point, after the 2.8% error was applied (clonal diversity, *R* = 0.58). Observed heterozygosity was 0.246 and in Hardy–Weinberg equilibrium, showing no evidence of inbreeding (*F*_IS_ = 0.016). Nucleotide diversity was moderate (*π* = 0.241), with 64 to 89% alleles shared among the eight multi-locus genotypes. This meadow had a high number of private alleles (*n* = 207).

**Figure 2.**
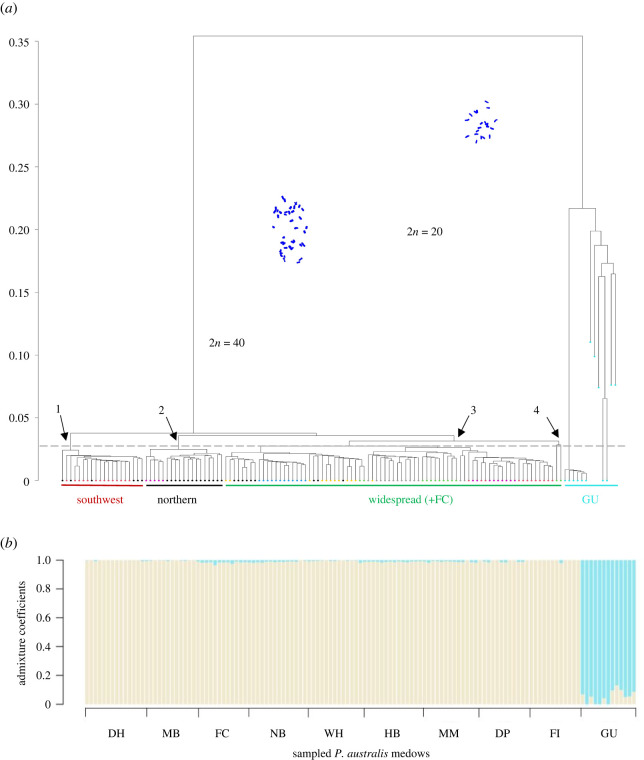
Synthesis of genomic diversity and structure among *Posidonia* meadows. (*a*) Phylogram showing the relationship among *Posidonia* samples, based on 18 021 SNPs. Broken line indicates the 2.8% cut-off for SNP calling error, as defined by technical replicates. Karyotypes are indicated along the branches, Guischenault Point clade (light blue closed circle) 2*n* = 20, all other sites are 2*n* = 40; clade 1 (southwest) Nanga Bay (black closed circle), White Island (brown closed circle); clade 2 (northern) Dirk Hartog Island (black closed circle), Herald Bight (purple closed circle); clade 3 (widespread) Middle Bluff (yellow closed circle), Fowlers Camp (green closed circle), Nanga Bay (black closed circle), Herald Bight (purple closed circle), Monkey Mia (grey closed circle), Dubaut Point (brown closed circle) and Faure sill (blue closed circle); clade 4 low-frequency genotype at Fowlers Camp (green closed circle). (*b*) Admixture results for optimal *K* ancestral populations, where *K* = 2. Each bar corresponds to an individual, with shared colour indicating genetic homogeneity. See table 1 for abbreviations.

A cladogram showed two distinct clades, one containing all samples from Guischenault Point (site 7) and a second containing all samples from the high heterozygosity meadows (all sites, except 7), and including the adjacent meadow to Guischenault Point at Herald Bight (site 6; figure 2*a*). The Guischenault Point clade contained all eight multi-locus genotypes, including one which was resampled on six occasions. The second clade contained multi-locus genotypes from all high heterozygosity meadows (nodes 1–4 indicated above the 2.8% error, figure 2*a*). Population admixture analysis supported *K* = 2 clusters (figure 2*b*; electronic supplementary material, figure S2*a*) for the complete dataset, and is consistent with two distinct clades in the cladogram. No significant structure was detected among the nine high heterozygosity meadows (*K* = 1; electronic supplementary material, figure S2*b*). The hierarchical AMOVA partitioned a significant amount of variation between Guischenault Point and all high heterozygosity meadows (between ploidy *F*_Sr_: 85.4%, *p* = 0.001; table 2). There was very little variation among meadows (*F*_ST_: 1.1%, *p* = 0.001) relative to within meadows (*F*_IS_: 13.5%, *p* = 0.001). Similar amounts of variation were attributed to variation among (*F*_ST_: 46.8%, *p* = 0.001) relative to within (*F*_IS_: 47.3%, *p* = 0.001) high heterozygosity meadows.

**Table 2.** Hierarchical AMOVA. Variance among *P. australis* meadows based on all 18 021 SNPs with 999 permutations.

source of variation	statistic	d.f.	sum sq	mean sq	sigma	%	*p*-value
all sampled meadows (*n* = 10):							
between ploidy	*F*sr	1	28957.1	28957.1	1218.4	85.4	<0.001
among meadows	*F*_ST_	1	1097.5	1097.5	15.8	1.1	0.001
within meadows	*F*_IS_	126	24234.4	192.3	192.3	13.5	0.001
total	*F*_IT_	128	54289.0	424.1	1426.6	100.0	
high heterozygosity meadows (*n* = 9):							
between gulfs	*F*sr	1	1166.8	1166.8	6.9	5.9	0.122
among meadows	*F*_ST_	7	5383.1	769.0	55.4	46.8	0.001
within meadows	*F*_IS_	107	5991.0	56.0	56.0	47.3	0.001
total	*F*_IT_	115	12540.8	109.1	118.3	100.0	

### (c) Ploidy and genome size

The karyotype of somatic chromosomes for plants from Guischenault Point indicated they were diploid (2*n* = 2*x* = 20; table 3 and figure 2*a*). Karyotypes from shoots in the high heterozygosity meadow at Middle Bluff were tetraploid (2*n* = 4*x* = 40). The genome size was estimated at 2C value of 4.56 pg ± 0.030 s.e. for the 2*n* = 20 karyotype from Guischenault Point, while the 2*n* = 40 karyotype from Middle Bluff had a 2C value of 7.89 pg ± 0.049 s.e. The polyploid genome was significantly larger at approximately 1.7× the diploid genome size (*t*-test; *p* < 0.001). The relative length and form percentage of chromosomes was significantly longer in diploid compared to polyploid karyotypes (relative length = 10.0 µm and 5.0 µm, *t*-test, *P* < 0.001; form percentage = 4.07 and 1.86, *t*-test, *p* < 0.001; electronic supplementary material, table S3). No significant differences were observed for the remaining six chromosome parameters.

**Table 3.** Ploidy and genome size for *P. australis* samples from two meadows.

population	ploidy level	2*n*	2C-value (pg ± s.e.)	1C-value (pg)	1C*x*-value (pg)	holoploid genome size (Mbp)	monoploid genome size (Mbp)
Guischenault Point (GU)	2*x*	20	4.56 ± 0.030	2.28	2.28	2229.84	2229.84
Middle Bluff (MB)	4*x*	40	7.89 ± 0.049	3.94	1.97	3853.32	1926.66

## 4. Discussion

We identified that *P. australis* meadows sampled across Shark Bay, Western Australia, consisted of a single polyploid clone spanning more than 180 km in fragmented, near-shore meadows. This makes it the most widespread known clone on earth. There was only one diploid meadow (Guischenault Point) among the 10 locations sampled. Whole-genome duplication associated with polyploidy appears to have enabled the *P. australis* clone to occupy new habitat and/or outcompete the diploid progenitor(s) within Shark Bay during rapid changes in environmental conditions following the Last Glacial Maximum. Shark Bay contains large areas of sandy sediment and is sheltered from oceanic swells. The shallow, sheltered, environment is ideal for clonal growth and vegetatively spreading meadows. Over millennia, shallowing coastal banks and sills from the biological capture of carbonate sediments have resulted in more extreme conditions throughout Shark Bay, with hypersalinity, extremely high light levels and wide temperature fluctuations in a phosphorus-limited system [38,40,41,43]. Our findings suggest a significant new example of polyploidy as a successful evolutionary strategy that enabled an advantage over diploid progenitor(s) and access to new, disturbed or harsher habitats as they developed [5,6,14]. The observed widespread distribution of the polyploid clone (greater than 180 km) is consistent with vegetative expansion following occupation of habitat created by inundation associated with a period of rapid sea-level rise approximately 8500 years ago [38]. The single *P. australis* clone exceeds that of an ancient diploid *Posidonia oceanica* clone discovered in the western Mediterranean that spans up to 15 km and may be greater than 100 000 years old [22]*.* Individual seagrass clones may persist almost indefinitely if left undisturbed, as they rely on vegetative, horizontal rhizome expansion, rather than sexual reproduction [25].

Our multiple lines of evidence are consistent with extensive polyploid meadows across Shark Bay. High heterozygosity probably confers a fitness advantage (heterosis) and may mask the effects of deleterious mutations. The highly negative *F*_IS_ values are the result of a whole-genome duplication event between different *Posidonia* lineages. The *H*_ind_/*H_E_* statistic for high heterozygosity samples was 1.0, where individual *H*_ind_/*H_E_* values were 3 to 4 times those of diploid individuals from Guischenault Point, and consistent with tetraploidy. The traditional estimation of population diversity statistics was compromised by clonality (low sample size as a result of resampling the same widespread clone). However, a direct comparison showed microsatellite loci identified 11 multi-locus genotypes (out of 28 shoot samples) at Guischenault Point and six (out of 27 shoot samples) at Fowlers Camp [27], compared to eight and two respectively, using 18 021 SNPs (this study), raising the question of whether SNPs are the best marker to study clonality.

The genome size of the polyploids was less than double (1.7×) that of the diploid individuals. Two explanations are plausible. This could be an older autopolyploid event in which there has been a significant loss of duplicated genes (rediploidization), or hybridization leading to allopolyploidy occurred in which the alternate progenitor had a smaller genome size than *P. australis*. All eight Australian *Posidonia* species have a somatic chromosome number 2*n* = 20 [64], along with the most basal member of the genus from the Mediterranean Sea, *P. oceanica* [65]. The genome size for *P. oceanica* (6.2 pg [28]) is considerably larger than *P. australis*, while the genome sizes for other Australian *Posidonia* species are currently unknown. Consequently, differentiating between these two competing hypotheses is challenging. However, based on the currently available evidence, it is less likely that autopolyploidy in edge of range meadows would lead to such a significant increase in genetic diversity. We propose that polyploidy as a result of hybridization between *P. australis* and an as yet unidentified *Posidonia* spp. was the most likely pathway. *Posidonia coriacea* and *P. angustifolia* would seem likely contenders, as they are also known from the Shark Bay region [43].

We showed that a single widespread polyploid clone spanned at least 180 km, from White Island to the Faure sill near L'Haridon Bight in the eastern gulf of Shark Bay. Horizontal vegetative expansion of the clone means the genetic signature of polyploidy was retained spatially and temporally. However, clones become fragmented as they age, so genetically identical ramets can be no longer physically connected through rhizomes. Thus, a direct measurement of clone size and age can be challenging [66]. We used the total estimated area of *P. australis* meadows in Shark Bay (200 km^2^ pre 2010/11 heatwave [43]), divided by a conservative range in average annual rhizome extension of 0.15–0.35 m year^−1^ based on *P. australis* from Oyster Harbour [67] and raised to an exponent factor of 2.5 to account for complex nonlinear rhizome branching [68] to estimate the time required for *P. australis* to reach its current range within Shark Bay. The derived estimate was a maximum of 4500 years old, a date which corresponds to the Holocene high stand for the region (up to 2.0 m above current sea level; [69]). The same calculations applied to the largest *P. oceanica* clone in the western Mediterranean Sea [22], which has a growth rate closer to 0.04 m year^−1^, put it at approximately 7900 years old. Similar calculations for a *Thalassia testudinum* clone indicate its predicted age was less than 6000 years [23].

Global records of large clones across four seagrass families (summarized in the electronic supplementary material, table S4) suggests they are effective at tracking sea-level changes. The one shared difference between these large, old clones (*P. oceanica* in the western Mediterranean [22], *Zostera marina* in the Baltic Sea [24,70], *Thalassia testudinum* in the Indian River Lagoon, Florida [23]) and our results here is that these previous studies showed no evidence for polyploidy, nor do they currently occur in extreme environments. Our data further support a hypothesis and evidence based on 900 angiosperm species, that polyploid clones reproduce mostly through vegetative, or clonal growth, whereas diploids prefer sexual reproduction [71]. Localized mass flowering events, however, have been observed at multiple locations across the range of polyploid *P. australis* clone including at Fowlers Camp, Denham, Big Lagoon, Dubaut Point (unpublished observations by the authors) and Red Hill Bay and Useless Loop in the western gulf (most with three or more microsatellite alleles; [26,27], Sinclair *et al*. unpublished data). Fruit containing viable embryos have been collected from only two locations in Shark Bay (Guischenault Point and Red Hill Bay), to date, and both have much lower outcrossing rates than higher latitude meadows [27]. Additional microsatellite alleles were also observed in 38% of embryos genotyped from the diploid maternal plants at Guischenault Point, suggesting that unreduced pollen from the nearby polyploid clone was pollinating flowers on diploid plants (i.e. polyploid pollen backcrossing to diploid *P. australis*). Further, we note that several stigmas examined from the flowering polyploid were deformed and unlikely to produce viable fruit. The widespread polyploid clone may be at least partially sterile, thus explaining the seedling recruitment bottleneck in Shark Bay [72]. Together, these observations are consistent with seagrass having the capacity for extensive clonal expansion in relatively stable locations, with a virtual absence of sexual reproduction.

Maximum sequence variation among our 116 polyploid *Posidonia* SNP profiles was 4%, of which 2.8% of this variation was attributed to genotyping errors determined by our technical replicate data. We then infer the residual 1.2% variation may represent an accumulation of somatic mutations in different parts of a single widespread clone, creating spatially arranged subclones. Somatic mutations are common in plants [73], however, their frequency in seagrass clones remains unclear, with only one example documented in *Zostera marina* [24,70]. No such mutations were reported as a source of variation in *P. oceanica* [22] or *Thalassia testudinum* [23] clones that occupy tens of kilometres. Our approach to sampling, as in these previous studies (i.e. [22,23]), was not designed to target physically connected ramets or quantify somatic mutations, nevertheless they provide a plausible explanation for variation among the subclones identified in this study.

Whole-genome duplication through polyploidy can be a particularly effective mechanism to increase diversity at a species range edge, where populations are often small, low in genetic diversity, and living at physiological limits [74]. Here, the polyploid probably had an advantage over diploids because (i) stressful conditions promoted polyploid formation, (ii) conditions were unsuitable for diploid progenitors to grow and (iii) polyploid had an increased capacity for genetic change through the larger genome and consequently adapted faster to changing environments [6]. *Posidonia australis* meadows in Shark Bay are at the northern extent of their distribution. The widespread polyploid clone experiences an annual temperature change from 17°C in winter to 30°C in summer and 20 psu variation in salinity over its geographic range relative to oceanic conditions for diploid meadows (35 ± 1 psu and less than 8°C annual temperature range), all under very high light intensities [43]. The Shark Bay findings are consistent with a broad range of (natural and synthesized) terrestrial polyploids that have shown increased tolerance to stress relative to their diploid progenitors (reviewed in [15]). The polyploid *P. australis* clone also showed a capacity to recover from an extreme climate event via vegetative growth [49]. The proposed superiority of this polyploid clone over diploid *P. australis* suggests vegetative material from the polyploid is best for the restoration of degraded meadows in Shark Bay. Exactly how the polyploid clone varies its response to local environmental conditions is unknown and the subject of further research, but its relative abundance suggests that it has evolved a resilience to variable and often extreme conditions that enable it to persist now and into the future.

## Data Availability

The SNP data files (with and without technical replicates) are available at https://doi.org/10.5061/dryad.0cfxpnw4p [75]. Electronic supplementary material is available online [76].
